# 100 Gb/s Silicon Photonic WDM Transmitter with Misalignment-Tolerant Surface-Normal Optical Interfaces

**DOI:** 10.3390/mi10050336

**Published:** 2019-05-22

**Authors:** Beiju Huang, Zanyun Zhang, Zan Zhang, Chuantong Cheng, Huang Zhang, Hengjie Zhang, Hongda Chen

**Affiliations:** 1 State Key Laboratory on Integrated Optoelectronics, Institute of Semiconductors, Chinese Academy of Sciences, Beijing 100083, China; chengchuantong@semi.ac.cn (C.C.); zhanghuanl@semi.ac.cn (H.Z.); woniudidi@semi.ac.cn (H.Z.); hdchen@semi.ac.cn (H.C.); 2 Tianjin Key Laboratory of Optoelectronic Detection Technology and System, Tianjin Polytechnic University, Tianjin 300387, China; 3 Optoelectronics Research Centre, University of Southampton, Southampton SO17 1BJ, UK; 4 School of Electronic and Control Engineering, Chang’an University, Xi’an 710064, China; zhangzan@semi.ac.cn (Z.Z.); 5 College of Materials Science and Opto-Electronic Technology, University of Chinese Academy of Sciences, Beijing 100049, China

**Keywords:** vertical grating coupler, WDM transmitter, optical interconnects, silicon photonics, silicon optical modulator

## Abstract

A 4 × 25 Gb/s ultrawide misalignment tolerance wavelength-division-multiplex (WDM) transmitter based on novel bidirectional vertical grating coupler has been demonstrated on complementary metal-oxide-semiconductor (CMOS)-compatible silicon-on-insulator (SOI) platform. Simulations indicate the bidirectional grating coupler (BGC) is widely misalignment tolerant, with an excess coupling loss of only 0.55 dB within ±3 μm fiber misalignment range. Measurement shows the excess coupling loss of the BGC is only 0.7 dB within a ±2 μm fiber misalignment range. The bidirectional grating structure not only functions as an optical coupler, but also acts as a beam splitter. By using the bidirectional grating coupler, the silicon optical modulator shows low insertion loss and large misalignment tolerance. The eye diagrams of the modulator at 25 Gb/s don’t show any obvious deterioration within the waveguide-direction fiber misalignment ranger of ±2 μm, and still open clearly when the misalignment offset is as large as ±4 μm.

## 1. Introduction

The volume of the digital information captured, created, or consumed each year is expected to grow from 538 exabytes to 5.0 zettabytes between 2012 and 2020, and it will double about every two and a half years [1]. To manage this amount of information, higher data transmission method is needed, especially in the data centers and high-performance computing (HPC) systems. Unfortunately, traditional electrical interconnects based on copper have been proved to be the bottleneck for growing demand of HPC systems and high-speed data center application because electrical input/output (IO) suffers from low bandwidth, high crosstalk, high latencies and attenuation. Optical interconnects are proposed to solve the bottleneck of electrical interconnects due to its low crosstalk, low latency, high bandwidth and high energy efficiency. Optical interconnects are emerging as a better solution over electrical interconnects for rack-to rack, board-to-board, chip-to-chip, and even on-chip data communication.

Silicon photonics based optical interconnects are good approach to obtain fully integrated photonic circuits with lower cost and higher integration level because of its compatibility with silicon microelectronic process. By taking advantages of the existing CMOS-VLSI infrastructure, silicon photonics is considered to be a promising and economical platform for the monolithic integration of various active and passive optoelectronic devices used in optical interconnects, such as high speed modulators [2,3,4,5,6], photodetectors [7,8,9], grating couplers [10] and optical splitters/combiners [11,12]. Low cost and large-scale integrated photonic circuits can be achieved by silicon photonic platform to realize optical interconnects [13,14]. Now, there are several silicon platforms, including Luxtera’s 130 nm Freescale platform, Mellanox’s 150 mm foundry, MPW services through IMEC and IME of Singapore, 130 nm SiGe BiCMOS production line at IHP in Frankfurt, and CEA-Leti.

Impressive works in the field of optical interconnects based on silicon photonics have been reported [13,14,15,16]. Silicon transmitters are important building blocks in silicon photonic interconnects. Several WDM and pulse-amplitude-modulation (PAM) transmitters are proposed to realize high-speed optical links [17,18,19] by different schemes. For these high-speed silicon transmitters based on silicon photonics, coupling a light effectively from a single mode fiber (SMF, typically with core diameter of 9 μm) into the silicon waveguide (typical dimensions of 220 nm × 450 nm) in an economical way is a big challenge. Grating couplers are widely used as the optical interfaces between the SMF and the submicron silicon waveguides. However, conventional grating couplers are usually designed for tilted-fiber coupling in order to reduce the second-order back reflection. The tilted coupling scheme will lead to several problems. Firstly, the alignment is more time-consuming because of angle tuning, and the mode matching efficiency usually deviates from the design due to the unavoidable mode expansion in the fiber–chip surface cavity. Secondly, a costly angle-polishing process is required for the fiber packaging. Compared to conventional GCs, perfectly vertical grating couplers (PVGCs) enable improved alignment accuracy and relaxed packaging complexity by avoiding angle alignment errors. Because of the advantages, PVGCs are more suitable to be applied in a multichannel communication network with fan-in/fan-out array, interfacing with a multicore fiber or fiber array with more strict requirements of alignment accuracy and coupling uniformity. 

In this paper, we present a 4 × 25 Gb/s ultrawide fiber misalignment-tolerant WDM transmitter based on the bidirectional grating couplers (BGCs)-based E-O modulator array and the microring multiplexer function. The BGCs exhibit the advantages of easy fabrication, perfectly vertical coupling and large misalignment tolerance, and potentially enable interfacing with a fiber array or flip-chip-integrated VCSEL laser array for low-cost photonic packaging. Benefiting from the characteristic of the BGCs, the WDM transmitter shows strong robustness to the variation of the fiber positions. Eye diagrams don’t show obvious deterioration within the waveguide-direction fiber misalignment ranger of ±2 μm, and still open clearly when the misalignment offset is as large as ±4 μm.

## 2. Structure and Principle

**Figure 1:**
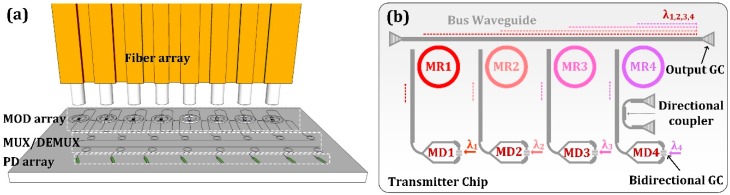
(**a**) Schematic diagram of the bidirectional grating coupler (BGC)-based wavelength-division-multiplex (WDM) optical interconnect tested with fiber array. (**b**) The schematic diagram of the proposed WDM transmitter.

As depicted in Figure 1a, a WDM optical interconnect circuit with an array of BGCs can be interfaced with a fiber array for rapid wafer-scale test and low-cost fiber packaging. We have demonstrated that a Mach–Zehnder-type E-O modulator can be built based on a BGC which functions as a 3 dB power splitter with symmetry grating design [20]. Therefore, it is experimentally feasible to realize a WDM transmitter integrated with a BGC-based modulator array and a microring (MR) array multiplexer, as schematically shown in Figure 1b. Four MZI modulators with embedded PN phase shifters in asymmetric arms are employed to generate optical modulation signals with different wavelengths. The MRs are designed with slightly different radii. Through the resonance coupling of the MR array, four optical signals can be uploaded to the bus waveguide and coupled out-of-plane with a standard GC. For wavelength alignment in measurement, each MR and MZI can be thermally tuned with integrated titanium nitride (TiN) heaters. In order to measure the optical spectra of MZI modulator and microring resonator independently, a directional coupler (DC) is inserted between the modulator and the microring to act as a −10 dB optical splitter.

### 2.1. Bidirectional Grating Coupler

The bidirectional grating coupler functions as the perfectly vertical grating coupler and power splitter in the Mach–Zehnder-type E-O modulator. According to the Bragg condition, the grating period *Λ* can be calculated by estimating the effective refractive index of the waveguide grating *N_effg_*, which can be expressed as:

(1) $$\Lambda =\lambda /N_{effg}$$

*λ* represents the vacuum wavelength. With the 2-D FDTD optimization, the optimal design parameters are grating period of 580 nm, etch depth of 70 nm, filling factor of 50%, period number of 22, respectively. For PVGCs, the cladding thickness have a big impact on the coupling performance as a well-designed cladding thickness can greatly reduce the upreflection loss. According to our simulations, the oxide cladding thickness of 1.3 µm offers an optimal performance with an in-plane coupling efficiency of 62% (−2.08 dB) and a 3 dB optical bandwidth of 80 nm. The upreflection power is suppressed to 16% around the center coupling wavelength, corresponding to an optical return loss of 8 dB. Although such a result is not as superior as that of a conventional GC, further improvement of the reflection loss can be achieved by utilizing an apodized grating design [21] or introducing a bilayer antireflection cladding [22]. 

**Figure 2:**
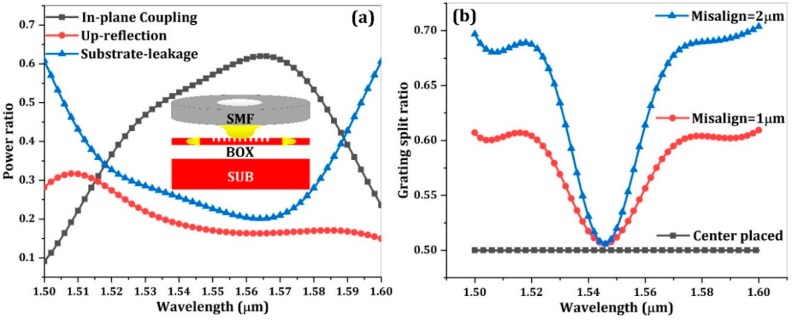
(**a**) The normalized optical power of different directions with perfectly vertical fiber incidence (inset picture shows the schematic of the BGC). (**b**) The wavelength-dependent grating split ratio with different fiber incident positions.

**Figure 3:**
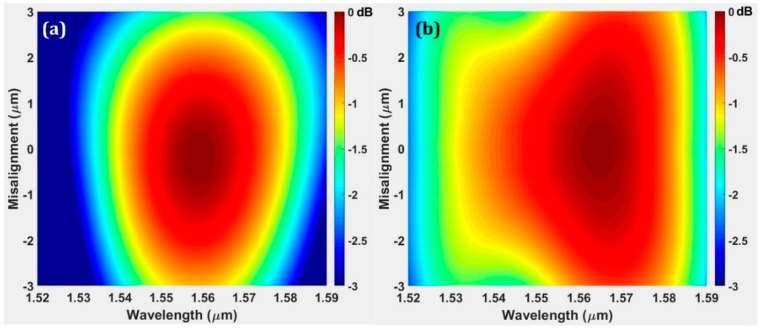
(**a**) The calculated coupling efficiency variation of a unidirectional grating coupler (GC) with fiber misalignment. (**b**) The calculated coupling efficiency variation of a bidirectional GC with fiber misalignment.

One notable advantage of the BGC is the large misalignment tolerance along the waveguide direction. For the BGC, the grating splitting behavior is of great significance to the BGC-based modulator operation. In order to investigate the influence of fiber misalignment, the grating split ratio (defined as the single arm power coupling divided by the total in-plane power coupling) with different fiber incidence positions are calculated with 2-D FDTD method. As shown in Figure 2b, the grating functions as a perfect 3 dB splitter with the fiber placed in the grating center due to the symmetry. With certain fiber displacement, the split ratio will increase significantly, which indicates the unbalanced power coupling in opposite directions. However, it is worth noting the split ratio is quite stable near the resonant wavelength of 1546 nm and nearly immune to fiber misalignment. This characteristic can be attributed to the wavelength-dependent second order reflection and would be very useful for modulator application. It allows a BGC-based modulator robustly working at a strong coupling wavelength in case of certain fiber misalignment. Apart from the splitting behavior discussions, the coupling efficiency variation with fiber misalignment is calculated for the BGC. For comparison, a unidirectional GC with same waveguide thickness and grating etch depth is also simulated. As shown in Figure 3, the BGC has a larger optical bandwidth and misalignment tolerance in the waveguide direction. The excess coupling loss caused by fiber misalignment is 0.95 dB (−3 µm misalignment) and 1.47 dB (3 µm misalignment) for unidirectional GC, while it is only 0.55 dB (±3 µm misalignment) for the bidirectional GC. 

### 2.2. BGC-Based Silicon E-O Modulator in Push-Pull Scheme

**Figure 4:**
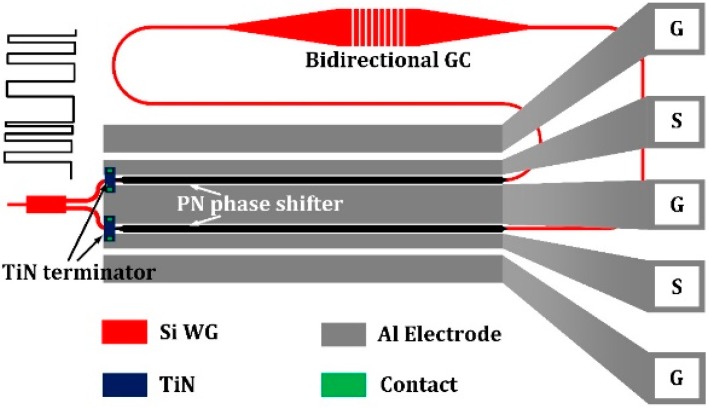
Schematic of demonstrated ultrawide misalignment tolerance modulator with bidirectional grating couplers.

The structural view of the bidirectional grating coupler-based optical modulator is shown in Figure 4, where the bidirectional grating coupler is used to couple light from the input fiber to the chip and split it into two arms. Comparing with traditional MZI modulators, our modulator avoids use of the conventional 3 dB beam splitter since the vertical grating coupler doubles as a fiber coupler and 3 dB optical splitter. Hence, the insertion loss of the modulator can be reasonably decreased.

Carrier-depletion-type optical modulators are utilized to develop our WDM transmitter chip because of their merits of high operation speed and low insertion loss. Both arms of the MZI contain a carrier-depletion-type phase shifter with length of 2 mm, cooperating with travelling wave electrode of GSGSG pattern to enable dual-port differential operation in a push-pull scheme. A low driving voltage signal with 2.5 V swing is utilized, which is CMOS-compatible and thus allows a direct integration with the electronic driver circuits. Low driving voltage can enable the use of most advanced CMOS technologies, which is beneficial for optimizing the energy efficiency. For example, the typical supply voltage for the feature size of 350 nm and 180 nm CMOS process is 3.3 V and 1.8 V. When the smaller feature size is chosen, higher speed and lower supply voltage can be obtained.

The arm length difference of the MZI is introduced to generate interference patterns in spectra similar to microring resonators. As the length difference between two arms is designed to be 248.4 μm, FSR of 2.4 nm can be obtained. The resonance behavior will enhance the modulation efficiency of MZI modulators. For asymmetrical MZI, the resonance condition is:

(2) $$\Delta \varphi =d\cdot N_{eff}\cdot \frac{2\pi }{\lambda }=\left(2m+1\right)\pi$$

Δ*φ* is the phase shift, *d* is the length difference between the two arms, *N_eff_* is the effective index of the single mode waveguide, *λ* is resonance wavelength, *m* is an integer. According to Equation (2), *d* is proportional to *λ*:

(3) Δd/d=Δλ/λ

Δ*d* and Δ*λ* are small variations of *d* and *λ* respectively. The optical phase change by Δ*d* is:

(4) $$\Delta \varphi =\Delta d\cdot N_{eff}\cdot \frac{2\pi }{\lambda }=2\pi \cdot d\cdot \Delta \lambda /\lambda^{2}\cdot N_{eff}$$

The effective refractive index of the phase shifter waveguide arms can be changed with bias voltage. The optical phase change due to bias voltage can be expressed as:

(5) $$\Delta \varphi =\Delta N_{eff}\cdot L\cdot \frac{2\pi }{\lambda }$$

Δ*N_eff_* is the change of the effective index. *L* is the length of MZI waveguide arms with embedded carrier-depletion-type phase shifter. When asymmetrical MZI resonances and destructive interference occurs, the optical phase change in Equation (4) and (5) is *π*. From Equation (4) and Equation (5), we can obtain:

(6) $$2\pi \cdot d\cdot \frac{\Delta \lambda }{\lambda^{2}\cdot N_{eff}}=\Delta N_{eff}\cdot L\cdot \frac{2\pi }{\lambda }$$

The equation can be further simplified as:

(7) $$\Delta N_{eff}=d\cdot \Delta \lambda /\lambda \cdot N_{eff}/L$$

For asymmetrical MZI, the FSR can be calculated as:

(8) $$\mathrm{FSR}=\lambda^{2}/(N_{g}\cdot d)$$

*N_g_* is the group refractive index of the single mode waveguide. The relationship between the FSR, Δ*λ* and Δ*φ* can be expressed by:

(9) Δφ=2π·Δλ/FSR

When phase shift is *π*, Δ*λ* is:

(10) $$\Delta \lambda =0.5\cdot \mathrm{FSR}=0.5\cdot \lambda^{2}/(N_{g}\cdot d)$$

According to Equations (7) and (10), we obtain:

(11) $$\Delta N_{eff}=\lambda /\left(2L\right)\cdot N_{eff}/N_{g}$$

Simulation results show that the effective index and group index for single mode rib waveguides of height 220 nm, width 500 nm and slab 60 nm is 2.52 and 4.03.

## 3. Experiment Results

**Figure 5:**
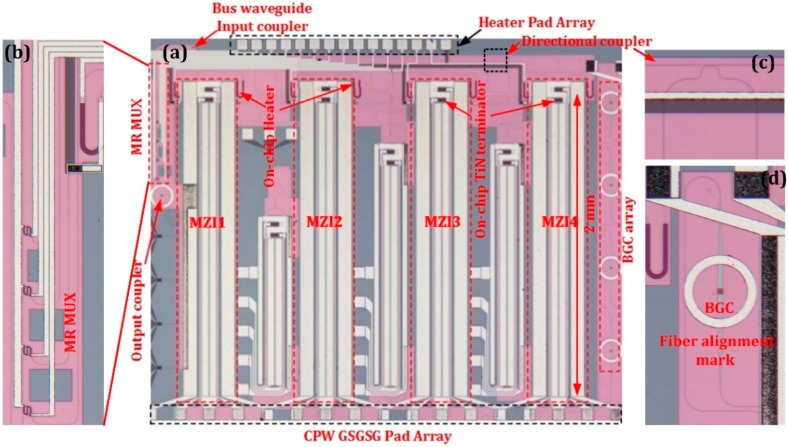
(**a**) Optical micrograph of the WDM transmitter with surface-normal optical interfaces. (**b**) The zoom-in picture of the MR Multiplexer. (**c**) The zoomed-in picture of the directional coupler. (**d**) The zoom-in picture of the bidirectional grating coupler-based surface-normal optical interface.

The four-channel WDM optical transmitter is realized with the silicon photonic MPW line of IME, Singapore. Figure 5a shows the optical micrograph of the WDM transmitter implemented in a 200 mm SOI wafer with a 2 μm-thick buried oxide layer and a 220 nm-thick top silicon layer. The zoom-in picture of the functions of the MR MUX, the DC and the BGC are respectively shown in Figure 5b–d. The phase shifters are based on a ridge waveguide structure with 500 nm width and 60 nm slab thickness. Four asymmetric MZI modulators with surface-normal optical interfaces are employed to generate modulated signal at different wavelengths. According to the plasma dispersion effect, holes contribute larger index change and less absorption than electrons. The width of the p-type doping region (300 nm) is set to be larger than the n-type doping region (200 nm). The p-type and n-type doping concentration are 7 × 10^17^ cm^−3^ and 5 × 10^17^ cm^−3^, the P^+^ and N^+^ regions are doped to a concentration of 10^20^ cm^−3^ to form low resistivity ohmic contact. Both arms of the MZI contain a 2 mm-long carrier-depletion type phase shifter, cooperating with GSGSG travelling wave electrode to enable dual-port low voltage differential operation. TiN resistors are integrated to form an on-chip terminator of 30 Ω to ensure impedance matching. Two thermal phase shifters are also incorporated within the interferometer to adjust the operation point at quadrature on the positive slope. Each MZI and microring resonator can be thermally tuned by an integrated heater implemented by a TiN resistor, so the working wavelengths of the modulators and the WDM function can be tuned to the same value. To make full use of the chip area, there are some other individual devices shown in the chip layout, such as three MZI modulators with different size and four Ge photodetectors.

### 3.1. Bidirectional Grating Coupler

**Figure 6:**
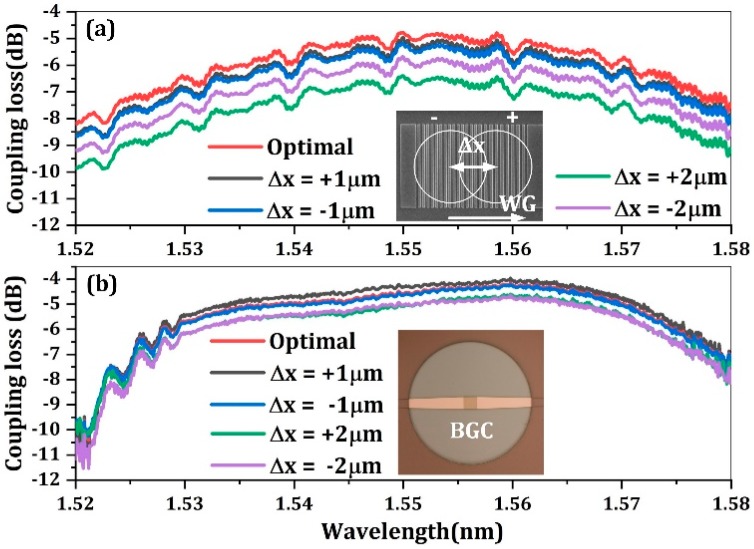
(**a**) The coupling efficiency spectra with waveguide-direction fiber misalignment for a conventional GC. (**b**) The coupling efficiency spectra with waveguide-direction fiber misalignment for a bidirectional GC.

To investigate the fiber misalignment tolerance of the BGC, a discrete BGC with balanced arms and MMI combiner is measured with a high-precision vertical fiber alignment system with a step resolution of 20 nm to ensure a small alignment error. For comparison, the misalignment tolerance of a conventional GC (utilized for output coupling interface) is also studied. Figure 6a,b show the coupling efficiency spectra response of the fiber incidence position variations within the range of ±2 µm for the conventional GC and BGC, respectively. The measurement result of the conventional GC is obtained with a back-to-back configuration, while the result of the BGC is calculated by normalizing the output coupler loss and the MMI insertion loss. When the fiber is tuned in the optimal position, the minimum coupling loss reaches −4.8 dB and −4 dB, respectively. The measured coupling loss of the two couplers are lower than the simulation results, which is possibly due to fabrication imperfections. Interestingly, although the simulation work indicates the two kind of couplers are close in peak coupling efficiency, the measured coupling efficiency of the BGC is higher than that of a conventional GC within the same photonic platform. This may be attributed to the excess coupling loss resulting from the free-space mode expansion within a tilted fiber experimental set-up. When the fiber is slightly tuned from the optimal position, the misalignment tolerance can be analyzed. The measured results show good accordance with the simulation results shown in Figure 3. The BGC has a more symmetric and larger misalignment tolerance along the waveguide direction. The excess coupling loss of the BGC is lower than 0.7 dB within a misalignment range of ±2 µm. However, the coupling deterioration of the conventional GC is as high as 1 dB and 1.68 dB with a fiber tuning of −2 µm and +2 µm, respectively. These experimental results clearly demonstrate the improvement of fiber misalignment tolerance with a BGC design.

### 3.2. BGC-Based E-O Modulator in Push-Pull Operation Mode

**Figure 7:**
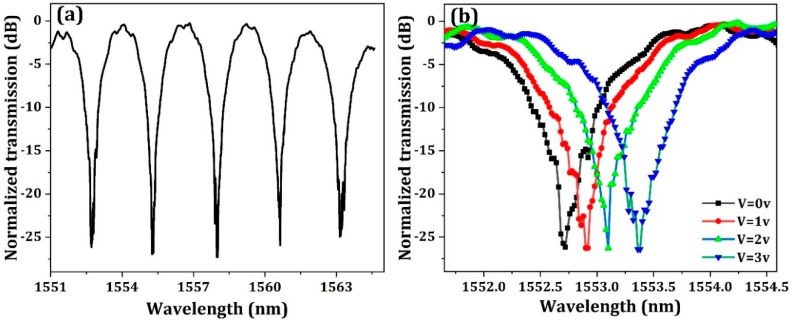
Static characteristics of the modulator: (**a**) Spectra response of modulator with 2.4 nm FSR, (**b**) Spectra response of modulator at different reversed voltages.

To investigate the optical spectrum of MZI modulator, 10% output optical power of the MZI modulator is coupled out by a directional coupler and connected with an additional output grating coupler. The measurement result is shown in Figure 7. As shown in Figure 7a, the measured FSR is about 2.5 nm, which is slightly larger than the designed value due to fabrication imperfection. As shown in Figure 7b, the transmission spectrum shifts about 0.8 nm with a bias voltage of 3 V, which corresponds to a *VπL* of 0.9 V·cm. The fiber-to-fiber optical loss is 11.2 dB, including the conventional grating coupling loss of 4.8 dB and the bidirectional grating coupling loss of 4 dB, the MMI combiner insertion loss of 0.8 dB and the phase shifter insertion loss of 1.6 dB. If the new coupling method is adopted and the conventional grating coupling loss can be possibly reduced to 1.5 dB [23], the fiber-to-fiber loss of the silicon modulator can be decreased to lower than 8 dB. High on-off extinction ratio of 27 dB is obtained for the MZI optical spectra, which indicates the near perfectly 3 dB power splitting behavior of the grating.

**Figure 8:**
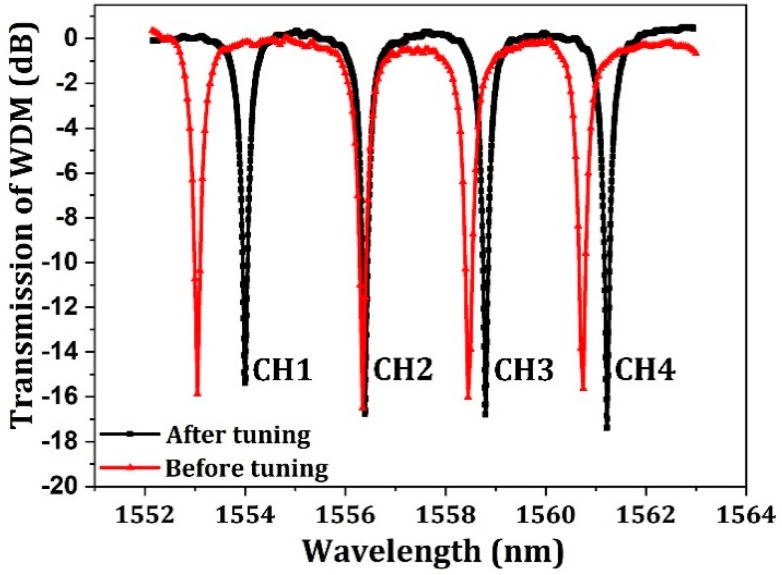
Normalized optical spectra of microring multiplexer before and after thermal tuning with a channel space of 2.4 nm.

Since the designed channel space is 2.4 nm, the FSR of 4 channels WDM should be larger than 9.6 nm. Thus, the radii of the microrings should be slightly smaller than 9.88 μm, and the radii of four microrings are designed to be slightly different to obtain WDM channel spacing of 2.4 nm. Figure 8 shows the normalized optical spectra of microring multiplexer before and after thermal tuning. The MR-based WDM obtains a uniform channel space of 2.4 nm after thermal tuning, and the overall tuning power was 12.54 mW.

**Figure 9:**
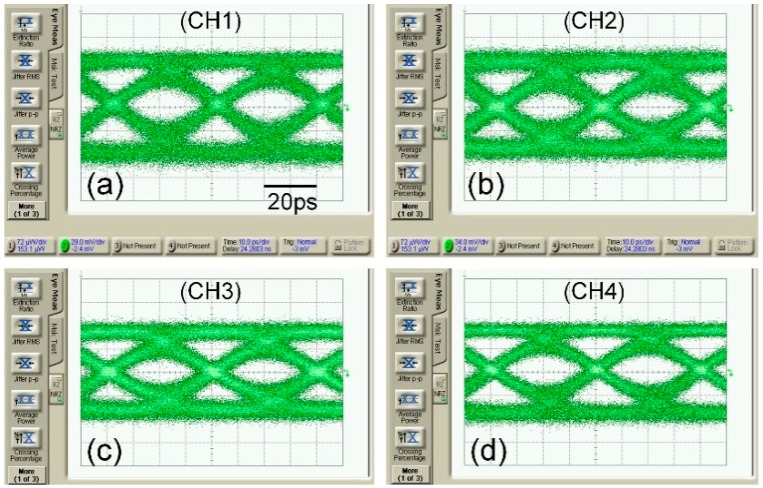
25 Gb/s eye diagram of WDM transmitter with different wavelengths: (**a**) 1554 nm, (**b**) 1556.4 nm, (**c**) 1558.8 nm, (**d**) 1561.2 nm.

The dynamic high frequency characteristics of the WDM transmitter were tested. Monochromatic light with wavelength of 1550 nm from a tunable laser is coupled to the chip by a polarization maintaining (PM) fiber. The high speed PRBS data stream generated by SHF 12104A is amplified by CENTELLAX OA4SMM4 microwave amplifier with a typical gain of 17 dB to get sufficiently high driving voltage swing. The output RF signal from microwave amplifier and DC bias are combined by Anritsu K250 to provide reversed DC bias, and then launched into the modulator using a 40 GHz microwave probe. The optical output of modulator is detected by high-speed photo-receiver 1474-A, and connected to an oscilloscope Agilent DCA 86100C for eye diagram observation. Figure 9 shows the eye diagram measurement results. With the differential RF driving signals at 25 Gb/s, we demonstrate that the optical output signals at all four channels have a clear eye opening with a healthy margin.

**Figure 10:**
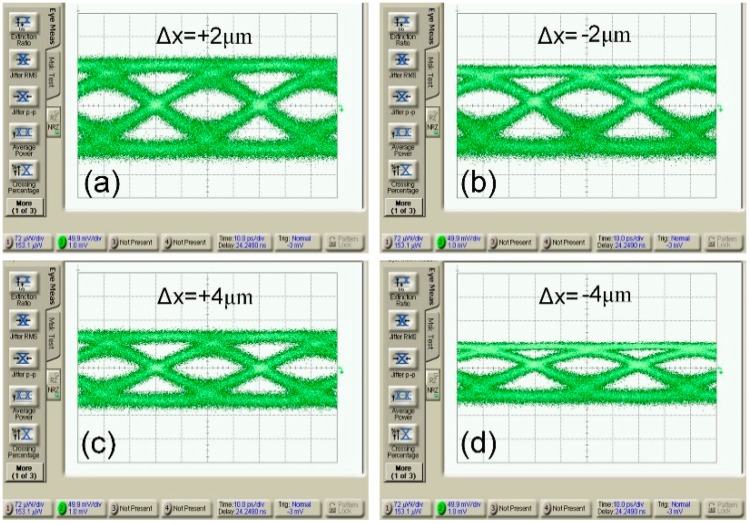
25 Gb/s eye diagram of WDM transmitter at different fiber offset from the central position of the grating coupler along the horizontal direction of waveguide: (**a**) +2 μm offset, (**b**) −2 μm offset, (**c**) +4 μm offset, (**d**) −4 μm offset.

The fiber misalignment tolerance on the modulator dynamic performance is investigated by tuning the fiber position along the waveguide direction. Eye diagrams with fiber misalignment along the waveguide direction are shown in Figure 10. From our simulation results of the grating splitting behavior, it is indicated that the modulator performance is more robust to fiber misalignment around the grating resonant wavelength of 1546 nm. However, the coupling efficiency of the bidirectional grating coupler reaches a maximum near 1560 nm. Therefore, we choose a working wavelength of 1554 nm as a trade-off between the optical loss and the grating splitting sensitivity. The eye diagrams don’t show any obvious deterioration within the waveguide direction misalignment range of ±2 μm, and still open clearly when the horizontal direction misalignment range is as large as ±4 μm. Table 1 shows the characteristic parameters of this work and the references. As comparing with references, the PVGCs-based modulator shows wider misalignment tolerance and reduced packaging difficulties by avoiding angle alignment, and has potential to provide low cost optical interconnects solutions.

## 4. Conclusions

We have experimentally demonstrated a 4 × 25 Gb/s WDM transmitter based on microring multiplexer and asymmetrical MZI modulator with surface-normal optical interface. According to our derivation, the modulation efficiency of the asymmetrical MZI modulator is higher than a symmetrical MZI modulator. Benefiting from higher modulation efficiency and a differential phase shifter of two arms, low voltage operation is achieved using a 2.5 V differential driving signal. By utilizing bidirectional grating couplers, the WDM transmitter shows ultrawide misalignment tolerance. The transmitter with ±4 μm fiber misalignment tolerance provides an attractive solution to the problem of optical coupling between fiber and waveguide. The vertical grating coupler-based transmitter has potential to provide low cost optical interconnect solutions and can serve as a photonic platform for developing high bandwidth optical interconnects and optical computing systems in the next generation of optical networks.

## Figures and Tables

**Table 1:** The characteristic parameters of this work and the references.

-	Ref [14]	Ref [18]	Ref [19]	This Work
**Scheme**	Ring WDM	Ring WDM	Ring DWDM	MZI WDM
**Speed**	10 Gb/s	12.5 Gb/s	10 Gb/s	25 Gb/s
**Channel Number**	4	4	5	4
**Channel Spacing**	0.8 nm	3.8 nm	0.5 nm	2.4 nm
***Vπ***	12 V	>3 V	>4 V	4.5 V
**Misalignment Tolerance**	-	-	-	±4 μm
